# Psychological Symptoms, Nutritional Risk, and Quality of Life in Hemodialysis Patients: A Structural Equation Modeling Study

**DOI:** 10.3390/ijerph23040475

**Published:** 2026-04-09

**Authors:** Tihomir Jovanović, Marin Mamić, Štefica Mikšić, Anđela Grgić, Jelena Tomac Jovanović, Ivana Mamić, Ivana Jelinčić, Hrvoje Vidić, Mirela Frančina, Harolt Placento, Ivan Vukoja, Božica Lovrić

**Affiliations:** 1 General Hospital Pakrac and Hospital of Croatian Veterans, Bolnička Ulica 74, 34550 Pakrac, Croatia; tihomir.jovanovic@ozbpakrac-bhv.hr (T.J.); jelena.tomac-jovanovic@ozbpakrac-bhv.hr (J.T.J.); 2 Faculty of Dental Medicine and Health Osijek, Josip Juraj Strossmayer University of Osijek, Crkvena 21, 31000 Osijek, Croatia; smiksic@fdmz.hr (Š.M.); ivana.mamic@pozeska-bolnica.hr (I.M.); jelincic.ivana@kbco.hr (I.J.); harolt.placento@obnasice.hr (H.P.); bozica.lovric@pozeska-bolnica.hr (B.L.); 3 Faculty of Medicine, Josip Juraj Strossmayer University of Osijek, 31000 Osijek, Croatia; agrgic@mefos.hr (A.G.); hrvoje.vidic@pozeska-bolnica.hr (H.V.); ivan.vukoja@pozeska-bolnica.hr (I.V.); 4 General County Hospital Požega, Osječka 107, 34000 Požega, Croatia; mirela.francina@pozeska-bolnica.hr; 5 Department of Physical Medicine and Rehabilitation, University Hospital Centre Osijek, Josipa Huttlera 4, 31000 Osijek, Croatia; 6 Department of Psychiatry, University Hospital Centre Osijek, Josipa Huttlera 4, 31000 Osijek, Croatia; 7 General Hospital Našice, Bana Jelačića 10, 31500 Našice, Croatia; 8 Faculty of Medicine, University of Rijeka, Braće Branchetta 20, 51000 Rijeka, Croatia; 9 Department of Nursing, University of Applied Sciences Ivanić-Grad, Moslavačka 13, 10310 Ivanić-Grad, Croatia

**Keywords:** hemodialysis, quality of life, depression, stress, mental health, nutritional risk, structural equation modeling

## Abstract

**Highlights:**

**Public health relevance—How does this work relate to a public health issue?**

- Patients undergoing hemodialysis face a substantial burden of psychological symptoms, nutritional vulnerability, and impaired quality of life.
- This study addresses an important public health issue by linking mental health, nutritional risk, and physical functioning in a high-risk chronic disease population.

**Public health significance—Why is this work of significance to public health?**

- Depressive symptoms and stress were associated with poorer mental health, whereas nutritional risk was independently associated with worse physical functioning.
- The findings identify mental health as a key pathway through which psychological symptoms may influence physical functioning in hemodialysis patients.

**Public health implications—What are the key implications or messages for practitioners, policy makers and/or researchers in public health?**

- Routine hemodialysis care should include systematic psychological screening and nutritional risk assessment.
- Integrated care models that combine nephrology, mental health, and nutritional support may help improve patient functioning and quality of life.

**Abstract:**

Patients undergoing hemodialysis often experience reduced quality of life, with psychological symptoms and nutritional risk representing important determinants of patient functioning. This study aimed to examine the relationships between depression, anxiety, stress, nutritional risk, mental health, and physical functioning in patients undergoing hemodialysis, with particular emphasis on the mediating role of mental health. A cross-sectional study was conducted among 199 patients receiving hemodialysis in five Croatian hospitals. Depression, anxiety, and stress were assessed using the DASS-42, quality of life using the SF-36, and nutritional risk using the NRS-2002. Associations between variables were examined using Spearman’s correlation coefficient, while structural equation modeling was used to analyze direct and indirect relationships among psychological symptoms, nutritional risk, mental health, and physical functioning. Depression and stress showed significant negative effects on mental health, while mental health showed a significant positive effect on physical functioning. Nutritional risk had a significant direct negative effect on physical functioning. Mental health significantly mediated the relationship between depression and stress and physical functioning. These findings indicate that psychological symptoms and nutritional risk are important determinants of functioning and quality of life in hemodialysis patients and support the need for an integrated care approach that includes regular psychological and nutritional screening.

## 1. Introduction

Chronic kidney disease is a significant public health problem associated with high morbidity and mortality [1]. At the final stage of chronic kidney disease, kidney replacement therapy is required, most often in the form of dialysis [2]. In Croatia, chronic kidney disease also represents an important healthcare burden, and patients requiring kidney replacement therapy are predominantly treated with dialysis, with hemodialysis being the most commonly used modality [3]. This highlights the importance of understanding factors that influence quality of life, functional status, and overall well-being in patients undergoing long-term hemodialysis. Hemodialysis and peritoneal dialysis have significantly extended the lives of patients, but long-term treatment also brings numerous physical, psychological, and social challenges that can substantially affect daily functioning and general well-being [4].

The chronic nature of the disease, the demanding treatment regimen, functional limitations and uncertainty related to the prognosis of the disease may contribute to the development of psychological difficulties in these patients. Psychological symptoms, especially symptoms of depression, anxiety and stress, are relatively common in patients on hemodialysis and represent an important factor that can affect their quality of life [5]. Research shows that symptoms of depression and anxiety are common among patients undergoing hemodialysis, with depressive symptoms estimated to occur in approximately one quarter of patients. A recent study has shown a high prevalence of symptoms of depression, anxiety and stress among hemodialysis patients and a strong association of these symptoms with poorer quality of life [6]. Psychological symptoms are also associated with poorer adherence to therapy, increased incidence of complications and more unfavorable health outcomes [7,8]. During hemodialysis treatment, complications such as extremity cramps and hypotension are common and may further increase patients’ stress and psychological burden.

Numerous studies have shown that patients on hemodialysis have a significantly impaired quality of life compared to the general population, especially in the domains of physical functioning and mental health. For example, recent studies indicate that patients on hemodialysis have significantly lower scores in most domains of quality of life compared to control groups and patients treated with other methods of renal replacement [9,10]. Quality of life is a multidimensional concept that encompasses the physical, psychological and social well-being of an individual. In patients with chronic kidney disease, it is recognized as an important indicator of health status and is associated with numerous clinical outcomes, including morbidity, mortality and frequency of hospitalization [11,12]. The quality of life of patients on hemodialysis can be influenced by a number of factors, including age, duration of hemodialysis, comorbidities, social circumstances and subjective perception of one’s own health status [13,14,15]. In the context of quality of life assessment, it is particularly important to distinguish between the mental and physical dimensions of health. Mental health is an important component of quality of life, encompassing emotional well-being, psychological stability, and an individual’s ability to cope with the daily stressors associated with illness and treatment. Mental health can play a significant role in the way patients perceive and assess their physical functioning. Previous research suggests that psychological symptoms such as depression and anxiety can negatively affect the perception of physical health and functional ability [5]. Therefore, it is assumed that mental health can act as a mediating factor in the relationship between psychological symptoms and physical functioning [4].

An important aspect of the health status of patients on hemodialysis is the state of nutrition. Malnutrition is a common complication in patients with chronic kidney disease and can occur as a result of reduced food intake, metabolic disorders, chronic inflammation and catabolic effects of dialysis [16,17]. Poor nutritional status is associated with increased morbidity, higher mortality and reduced functional ability of patients [18,19]. During hemodialysis treatment, amino acids and other nutrients are lost, while metabolic changes associated with uremia and inflammation further worsen nutritional status. As a result, patients on hemodialysis are at increased risk of malnutrition, which can have a negative impact on their general health and quality of life [20,21]. Recent evidence also suggests that poorer nutritional status and lower protein intake may be associated with reduced muscle mass, poorer physical functioning, and worse health-related outcomes in hemodialysis patients [22,23,24].

Previous studies have shown that psychological symptoms and nutritional status disturbances are common in hemodialysis patients and are associated with poorer quality of life. However, these factors have often been examined separately, or only in relation to selected quality-of-life domains. As a result, their simultaneous relationships and potential indirect pathways remain insufficiently understood. A clearer understanding of these relationships may help identify clinically relevant targets for integrated care aimed at preserving both mental and physical functioning in hemodialysis patients. Structural equation modeling provides an appropriate framework for examining such direct and indirect relationships.

Therefore, the aim of this study was to examine the relationships between depression, anxiety, stress and nutritional risk and the dimensions of quality of life in hemodialysis patients, with special emphasis on the role of mental health as a mediating variable in the relationship between psychological symptoms and physical functioning.

## 2. Materials and Methods

A cross-sectional study was conducted among 199 patients undergoing hemodialysis. The study was carried out from February 2025 to February 2026 in five Croatian hospitals: General County Hospital Požega, General Hospital Dr. Josip Benčević Slavonski Brod, General Hospital Pakrac and Hospital of Croatian Veterans, University Hospital Centre Zagreb, and University Hospital Centre Osijek. Ethical approval was obtained from the ethics committees of all participating institutions. Participants were recruited during their regular hemodialysis sessions. Patients who met the inclusion criteria were approached by the researchers or authorized healthcare staff and informed orally and in writing about the purpose of the study. They were explicitly informed that participation was voluntary and that refusal to participate or withdrawal from the study would not affect their treatment in any way. Questionnaires were completed anonymously in order to ensure confidentiality and reduce potential response bias. The study included adult patients with chronic kidney disease treated with hemodialysis who were able to speak and understand Croatian. Exclusion criteria included severe cognitive disorganization, impaired vision and/or hearing that prevented questionnaire completion, low literacy skills, unconsciousness, and the presence of cancer [25] or hyperthyroidism [26]. Patients with cancer and hyperthyroidism were excluded because both conditions may independently affect nutritional status, psychological symptoms, and quality of life, which could confound the relationships examined in this study. There were 116 men (58.3%) and 83 women (41.7%) in the sample. The mean age of the participants was 63.61 ± 14.88 years, and the average duration of dialysis treatment was 5.58 ± 6.71 years.

### 2.1. Instruments

The DASS-42 questionnaire was used to assess depression, anxiety and stress [27,28]. This instrument was selected because it is widely used for assessing symptoms of depression, anxiety, and stress and enables separate evaluation of these related but distinct psychological domains. The questionnaire was developed on a non-clinical sample of adolescents and adults and consists of 42 items divided into three subscales: depression, anxiety and stress, with each subscale containing 14 items. The depression subscale refers to the symptoms of dysphoria, hopelessness, self-devaluation, apathy and lack of interest, the anxiety subscale refers to the excitation of the autonomic system and situational anxiety, while the stress scale includes indicators of chronic, non-specific arousal, difficulty relaxing, agitation and impatience. Respondents answer on a Likert scale from 0 to 3, and the total score is formed as a linear combination of assessments by individual subscales. In this study, internal consistency was very high for depression (ω = 0.92) and stress (ω = 0.89) and good for anxiety (ω = 0.79). Regarding the validity of the subscales, the authors of the scale determined the correlation of the anxiety subscale with the Beck anxiety inventory of 0.81 and the correlation of the depression subscale with the Beck depression scale of 0.74 [28].

The SF-36 (Short Form Health Survey-36) questionnaire [29,30,31] was used to assess the quality of life. This instrument was selected because it is one of the most widely used generic measures of health-related quality of life and allows separate assessment of physical and mental health dimensions relevant to this study. It is a generic and frequently used instrument that consists of 36 items and assesses the subjective sense of health through eight dimensions: physical functioning, limitations in the realization of life roles due to physical difficulties, physical pain, perception of general health, vitality, social functioning, limitations in the realization of life roles due to emotional difficulties and mental health. The internal consistency of the physical functioning dimension from the SF-36 questionnaire, estimated by the McDonald omega coefficient (ω), was ω = 0.97, while for the mental health dimension it was ω = 0.83.

Nutritional risk was assessed using the NRS-2002 (Nutrition Risk Screening 2002) questionnaire [32,33,34]. This instrument was selected because it is a recommended and clinically applicable screening tool for identifying nutritional risk in hospitalized adult patients. It is one of the recommended tools for nutritional risk screening in hospitalized patients. The questionnaire includes an assessment of nutritional status, reduced food intake, weight loss, body mass index and disease severity. An overall score of less than 3 indicates the absence of a risk of malnutrition, while a score of 3 or more indicates a nutritional risk and the need for nutritional support. The internal consistency of NRS-2002 was estimated by the McDonald omega coefficient (ω) at the level of its components (nutritional status, disease severity and age) and amounted to ω = 0.71, whereby the obtained value should be interpreted with caution given that it is a clinical screening index and not a homogeneous psychometric scale. The instrument is easy to use and has a good predictive value for assessing nutritional status [35,36,37].

### 2.2. Statistical Methods

The normality of the distribution of numerical variables was assessed using the Kolmogorov–Smirnov test, together with inspection of skewness and kurtosis values. The Kolmogorov–Smirnov test indicated statistically significant departures from normality for most analyzed variables (*p* < 0.05), and notable deviations in skewness and kurtosis were particularly observed for depression, anxiety, and stress scores. Therefore, non-parametric methods were applied in the correlation analyses.

Associations between psychological variables, nutritional risk, and quality-of-life dimensions were examined using Spearman’s rank correlation coefficient (ρ), because the assumptions for parametric correlation analysis were not met.

Structural equation modeling (SEM) was used to examine the relationships among depression, anxiety, stress, nutritional risk, and quality-of-life dimensions. Because several observed variables showed deviations from normality, the model was estimated using the robust maximum likelihood estimator (MLR), with robust standard errors. Missing data were handled within the MLR framework using all available information, under the assumption that data were missing at random.

In the initial phase, a theoretically specified model including all hypothesized direct paths among the observed variables was tested. After estimation of the initial model, statistically non-significant paths were removed in order to obtain a more parsimonious final model, while preserving theoretically meaningful relationships.

Model fit was evaluated using several goodness-of-fit indices: the Comparative Fit Index (CFI), Tucker–Lewis Index (TLI), Root Mean Square Error of Approximation (RMSEA), and Standardized Root Mean Square Residual (SRMR). Values of CFI and TLI ≥ 0.90 were considered indicative of acceptable fit, whereas values ≥ 0.95 indicated good fit. For RMSEA and SRMR, values < 0.08 were considered acceptable, and values < 0.05 indicated good fit.

Internal consistency of the scales was evaluated using McDonald’s omega coefficient, with values of ≥0.70 considered acceptable, ≥0.80 good, and ≥0.90 excellent [38]. Statistical significance was set at *p* < 0.05.

Multicollinearity among predictors was assessed using variance inflation factors (VIF) and tolerance values. VIF values below 5 and tolerance values above 0.20 were considered acceptable, indicating no relevant multicollinearity. In the present study, VIF values ranged from 1.035 to 1.464, confirming the absence of multicollinearity. Autocorrelation of residuals was assessed using the Durbin–Watson statistic, with values close to 2 indicating no substantial autocorrelation. In the present analysis, the Durbin–Watson value was 2.044, suggesting no significant residual autocorrelation.

Post hoc statistical power was additionally estimated for the main indirect (mediation) effects in the structural model based on the observed standardized path coefficients and sample size. The estimated power was approximately 0.96 for the indirect effect of depression on physical functioning through mental health and approximately 0.80 for the indirect effect of stress on physical functioning through mental health, indicating that the achieved sample size was adequate for detecting the main mediation effects in the model.

All analyses were performed using JASP software, version 0.96.0 [39].

## 3. Results

Descriptive indicators of the investigated variables are shown in Table 1. The mean value of physical functioning according to SF-36 was M = 48.51 (SD = 33.14), while the mental health value was M = 58.67 (SD = 17.41). The mean scores for anxiety, depression, and stress were 5.45 ± 4.36, 3.45 ± 4.95, and 5.48 ± 5.72, respectively, with noticeable variability across participants. The average nutritional risk estimated by the NRS-2002 questionnaire was 1.84 ± 0.88 (Table 1).

The model showed mixed fit indices. While CFI and SRMR suggested acceptable fit, TLI and RMSEA indicated less satisfactory model fit. The chi-square test was statistically significant (χ^2^(3) = 11.32; *p* = 0.010). The comparative Fit Index was CFI = 0.911, while the Tucker–Lewis index was TLI = 0.733. The RMSEA value was 0.118 (90% CI 0.050–0.196), and the SRMR was 0.038. The values of the information criteria were AIC = 3586 and BIC = 3612 (Table 2).

In order to examine the association between psychological variables, nutritional risk and quality of life dimensions, Spearman’s correlations were used. Physical functioning was moderately positively associated with mental health (ρ = 0.373, *p* < 0.001) and negatively associated with anxiety (ρ = −0.187, *p* = 0.008), depression (ρ = −0.249, *p* < 0.001) and nutritional risk (ρ = −0.349, *p* < 0.001). Mental health was negatively associated with depression (ρ = −0.328, *p* < 0.001), stress (ρ = −0.262, *p* < 0.001), and nutritional risk (ρ = −0.163, *p* = 0.021). Anxiety, depression and stress were positively correlated with each other (Table 3).

**Figure 1:**
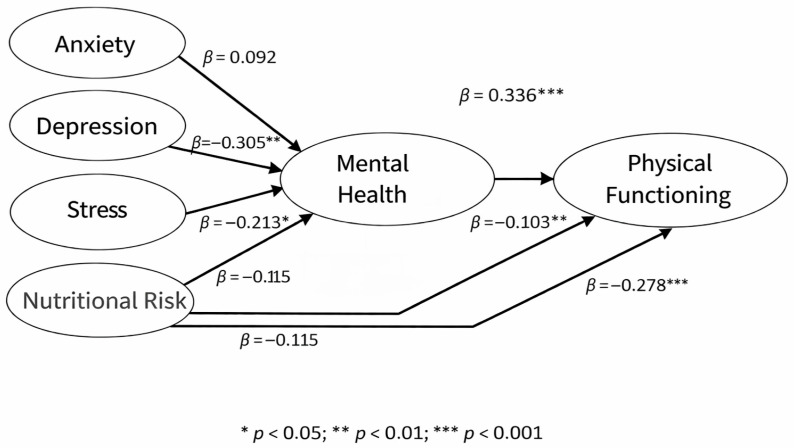
Structural model of the relationship between depression, anxiety, stress, nutritional risk, mental health, and physical functioning in hemodialysis patients.

Standardized regression coefficients are shown in Table 4. Depression (β = −0.305; *p* < 0.001) and stress (β = −0.213; *p* = 0.001) showed a statistically significant negative effect on mental health. Anxiety (*p* = 0.167) and nutritional risk (*p* = 0.083) were not statistically significant predictors of mental health. Mental health showed a statistically significant positive effect on physical functioning (β = 0.336; *p* < 0.001). Nutritional risk showed a statistically significant negative direct effect on physical functioning (β = −0.278; *p* < 0.001). The model explained 17.8% of the variance in mental health (R^2^ = 0.178) and 21.8% of the variance in physical functioning (R^2^ = 0.218) (Table 4, Figure 1).

The results of the indirect effects analysis are presented in Table 5. Mental health significantly mediated the relationship between depression and physical functioning (β = −0.103; *p* < 0.001) and between stress and physical functioning (β = −0.072; *p* = 0.002) (Table 5).

The overall effects of the variables included in the model are shown in Table 6. Depression showed a statistically significant negative overall effect on mental health (β = −0.305, *p* < 0.001) and on physical functioning (β = −0.103, *p* < 0.001). Stress also showed a significant overall effect on mental health (β = −0.213, *p* = 0.001) and physical functioning (β = −0.072, *p* = 0.002). Nutritional risk showed a statistically significant negative overall effect on physical functioning (β = −0.316, *p* < 0.001), while mental health showed a statistically significant positive effect on physical functioning (β = 0.336, *p* < 0.001) (Table 6).

## 4. Discussion

In this study, we examined the relationships between depression, anxiety, stress and nutritional risk, as well as the dimensions of quality of life in hemodialysis patients, with special emphasis on the role of mental health as a mediating variable in the relationship between psychological symptoms and physical functioning. The results indicate a significant correlation between psychological symptoms such as depression and stress, nutritional risk and quality of life dimensions in hemodialysis patients. Depression and stress showed a statistically significant negative effect on mental health. On the other hand, mental health showed a statistically significant positive effect on physical functioning. Such findings are in line with previous research [40] and indicate the important role of psychological factors in shaping the subjective assessment of health status in patients on hemodialysis. Nutritional risk has also been found to have a significant negative direct effect on bodily functioning, which is consistent with previous research [41]. This result indicates the importance of nutritional status in preserving physical functionality in this patient population. The results suggest that mental health may act as a mediating factor in the relationship between psychological symptoms and physical functioning. This means that symptoms of depression and stress can affect the physical functioning of patients partly through the deterioration of mental health [42], which highlights the complex interconnection of psychological and physical aspects of health in patients on hemodialysis and indicates the need for a holistic approach in their care [5].

Depression and stress have been found to have a significant negative effect on mental health. It is interesting to note that depression has a stronger negative effect on mental health than stress, which has proven to be a significant, but weaker, predictor. Patients on hemodialysis are exposed to emotional stress for a long time. Symptoms of depression can better reflect chronic psychological exhaustion than acute tension and shape the mental component of quality of life more strongly. Other studies also state that depression is the most common mental disorder associated with a poorer assessment of quality of life as a component of mental health in the population of patients undergoing hemodialysis [6,43]. These results support the justification of routine screening for psychological symptoms, which may be more useful than relying solely on the general clinical impression of the patient’s mental state.

Anxiety has not been shown to be a significant predictor of mental health in either direct or overall effects. This result can be partly explained by the statistical possibility that the effect of anxiety decreased after the simultaneous inclusion of depression and stress in the model. Anxiety symptoms in the life of hemodialysis patients are sometimes more situational and transient, while depression and chronic stress are more stable indicators of the load. The finding does not necessarily contradict the literature but shows that in this sample, anxiety did not have an independent predictive value after controlling for other psychological symptoms. Although anxiety has not been shown to be an independent predictor of mental health in this study, previous research shows that anxiety symptoms in hemodialysis patients may still be associated with poorer quality of life [13,44]. However, the intensity of this relationship is not always the same, and it is possible that the effect of anxiety is more sensitive to other clinical and sociodemographic factors, which is why it may not always retain statistical significance in multivariate models.

Mental health showed a statistically significant positive effect on physical functioning. Practically speaking, these results suggest that hemodialysis patients who have better mental health also function better physically. A 2025 study states that hemodialysis strongly affects both physical and mental health, and that psychological symptoms are associated with a lower quality of life [45]. Research on emotional intelligence in chronic hemodialysis further supports the idea that psychological processes affect both physical and mental state [46]. Our results support the understanding that the mental and physical components of quality of life are not separate entities but interrelated aspects of the experience of health.

Nutritional risk showed a statistically significant negative direct effect on physical functioning, while the effect on mental health was absent. Poorer nutritional status is likely to be reflected first in physical strength, muscle mass, endurance and daily functionality, while its relationship with mental health may be weaker or mediated by other factors. The results obtained are in line with previous studies that have shown that malnutrition is a strong predictor of mortality among dialysis patients [22,41]. These findings are consistent with previous studies showing that poorer nutritional status is associated with reduced physical functioning and worse health-related outcomes in hemodialysis patients [22,23,24]. In this study, the NRS-2002 Nutritional Status Assessment Questionnaire proved to be a tool by which, in addition to the assessed nutritional status, we identified patients with a higher risk of poor physical functioning as a dimension of quality of life.

Nutritional risk did not prove to be a statistically significant predictor of mental health in the final model, although a weak negative association was found between these variables in the correlation analysis. Although some studies have shown that poorer nutritional status is associated with both emotional and general aspects of quality of life, our results suggest that in this patient population, its effect is more strongly manifested on physical than on mental functioning [41]. It is possible that the association between nutritional risk and mental health can be seen in simpler analyses due to the general deterioration of health status, but that after the inclusion of depression and stress, nutritional risk no longer retains a sufficiently strong independent contribution to the explanation of mental health.

Mental health has been shown to significantly mediate the relationship between psychological symptoms and physical functioning. In other words, the overall effects of depression on physical functioning and stress on physical functioning stem from depression and stress worsening mental health, and poorer mental health then worsens physical functioning. This finding supports the assumption that mental health may represent an important mediating factor through which psychological symptoms may be related to the physical functioning of patients on hemodialysis. Although depression and stress did not have direct pathways to physical functioning in the final model, their overall effects were statistically significant due to indirect effects through mental health. Although not all papers test formal mediation, a meta-analysis of psychosocial interventions shows that reducing depression and anxiety leads to improved quality of life in hemodialysis patients, which indirectly supports this mediation model [47]. Clinically speaking, this means that depression and stress should not only be viewed as emotional problems but also as factors that can have wider consequences on the patient’s daily functionality.

### 4.1. Clinical Implications

The clinical application of these findings requires a shift towards integrated care. Since psychological symptoms such as stress and depression are associated with poorer physical functioning, regular screening is warranted. Nurses, as the most present team members, play a crucial role in the early identification of these changes. The same weight is carried by timely nutritional assessment, which serves as a key indicator of future physical functionality. Ultimately, the results support a treatment model in which psychological support and nutritional care are equal components of standard nephrology therapy.

### 4.2. Limitations of the Study

Despite the contribution of this research, it is necessary to take into account several methodological limitations. Primarily, the cross-sectional design of the study makes it impossible to draw definitive cause-and-effect conclusions; the correlations we found offer insight into the correlation of variables, but not into their precise time sequence. The question of representativeness also requires caution. Although the sample of 199 respondents from five Croatian hospitals represents a solid basis, its geographical orientation requires restraint in generalizing the findings to the entire patient population in Croatia or international contexts. Furthermore, although we used validated high-reliability instruments, such as the SF-36 and DASS-42 questionnaires, the data rely on patient self-assessment, which makes them susceptible to subjective conditions at the time of the interview. Statistical processing also reflects the specificities of this population; significant deviations from the normal distribution required the application of nonparametric methods and robust estimators in structural modeling. This variability suggests that results should be approached with analytical caution. Finally, although the structural model confirms significant relationships, part of the variance in mental health and physical functioning remains unexplained, which clearly indicates the presence of external factors that were not covered by this model but deserve attention in future research. The structural model should also be interpreted with caution because not all fit indices indicated satisfactory model fit, suggesting that the examined relationships may not be fully captured by the final model.

## 5. Conclusions

The results of this study suggest that depression, stress and nutritional risk are important determinants of quality of life in patients on hemodialysis. In this sample, depression and stress were associated with poorer mental health, while better mental health was associated with better physical functioning. Nutritional risk showed an independent negative effect on physical functioning, which further confirms the importance of timely recognition of malnutrition in this population. Also, mental health has been shown to be an important mediating factor in the relationship between depression and stress and physical functioning, which indicates the interconnection of psychological and physical aspects of health. The findings of the study support the need for an integrated approach to care in which, in addition to standard nephrological treatment, psychological symptoms and nutritional risk are systematically monitored in order to preserve the functionality and quality of life of patients on hemodialysis.

## Figures and Tables

**Table 1:** Descriptive indicators of the examined variables (*N* = 199).

	Min	Max	M ± SD
SF-36 Physical Functioning	0	100	48.51 ± 33.14
SF-36 Mental Health	8	100	58.67 ± 17.41
DASS-42 Anxiety	0	25	5.45 ± 4.36
DASS-42 Depression	0	30	3.45 ± 4.95
DASS-42 Stress	0	26	5.48 ± 5.72
NRS-2002 Overall Score	1	5	1.84 ± 0.88

Note: M is the arithmetic mean. SD—standard deviation; SF-36—Short Form Health Survey-36; DASS-42—Depression Anxiety Stress Scales-42; NRS-2002—Nutrition Risk Screening 2002.

**Table 2:** Structural model fit indices (*N* = 199).

Indicator	Value
χ^2^	11.32
df	3
*p*	0.010
CFI	0.911
TLI	0.733
RMSEA	0.118
RMSEA 90% CI	0.050–0.196
SRMR	0.038
AIC	3586
BIC	3612

Note: χ^2^—chi-square model fit test; df—degrees of freedom; CFI—Comparative Fit Index; TLI—Tucker–Lewis Index; RMSEA—Root Mean Square Error of Approximation; SRMR—Standardized Root Mean Square Residual; AIC—Akaike Information Criterion; BIC—Bayesian Information Criterion.

**Table 3:** Association between psychological variables, nutritional risk and quality of life dimensions (*N* = 199).

		1	2	3	4	5	6
1. SF-36 Body Functioning		—					
2. SF-36 Mental Health	ρ *p*	0.373 <0.001	—				
3. DASS-42 anxiety	ρ *p*	−0.187 0.008	−0.076 0.283	—			
4. DASS-42 depression	ρ *p*	−0.249 <0.001	−0.328 <0.001	0.302 <0.001	—		
5. DASS-42 Stress	ρ *p*	0.021 0.767	−0.262 <0.001	0.317 <0.001	0.279 <0.001	—	
6. NRS-2002 Overall Score	ρ *p*	−0.349 <0.001	−0.163 0.021	0.124 0.080	0.134 0.060	−0.015 0.828	—

Note: ρ—Spearman’s correlation coefficient; *p*—statistical significance.

**Table 4:** Standardized regression coefficients of the structural model (*N* = 199).

		β	SE	z	*p*	95% CI Lower	95% CI Upper
SF-36 Mental Health	Depression	−0.305	0.062	−4.888	<0.001	−0.428	−0.183
	Anxiety	0.092	0.067	1.381	0.167	−0.039	0.223
	Stress	−0.213	0.065	−3.255	0.001	−0.341	−0.085
	Nutritional risk	−0.115	0.066	−1.734	0.083	−0.245	0.015
SF-36 Physical Functioning	Mental health	0.336	0.061	5.544	<0.001	0.217	0.455
	Nutritional risk	−0.278	0.058	−4.795	<0.001	−0.391	−0.164

Note: β—standardized regression coefficient; SE—standard error; CI—confidence interval; *p* < 0.05 was considered statistically significant.

**Table 5:** Indirect effects in the structural model (*N* = 199).

	β	SE	z	*p*	95% CI Lower	95% CI Upper
Depression → mental health → physical functioning	−0.103	0.030	−3.405	<0.001	−0.162	−0.044
Anxiety → mental health → physical functioning	0.031	0.023	1.347	0.178	−0.014	0.076
Stress → mental health → physical functioning	−0.072	0.023	−3.139	0.002	−0.116	−0.027
Nutritional risk → mental health → physical functioning	−0.039	0.024	−1.606	0.108	−0.086	0.009

Note: β—standardized regression coefficient; SE—standard error; CI—confidence interval; *p* < 0.05 was considered statistically significant.

**Table 6:** Overall effects of variables in the model (*N* = 199).

	β	SE	z	*p*	95% CI Lower	95% CI Upper
Depression → Mental Health	−0.305	0.062	−4.888	<0.001	−0.428	−0.183
Depression → physical functioning	−0.103	0.030	−3.405	<0.001	−0.162	−0.044
Anxiety → mental health	0.092	0.067	1.381	0.167	−0.039	0.223
Anxiety → physical functioning	0.031	0.023	1.347	0.178	−0.014	0.076
Stress → mental health	−0.213	0.065	−3.255	0.001	−0.341	−0.085
Stress → physical functioning	−0.072	0.023	−3.139	0.002	−0.116	−0.027
Nutritional risk → mental health	−0.115	0.066	−1.734	0.083	−0.245	0.015
Nutritional risk → physical functioning	−0.316	0.058	−5.471	<0.001	−0.430	−0.203
Mental health → physical functioning	0.336	0.061	5.544	<0.001	0.217	0.455

Note: β—standardized overall effect; SE—standard error; CI—confidence interval; *p* < 0.05 was considered statistically significant.

## Data Availability

The data presented in this study are available on request from the first or corresponding authors.
